# Synthesis and Thermophysical Characterization of Fatty Amides for Thermal Energy Storage

**DOI:** 10.3390/molecules24203777

**Published:** 2019-10-21

**Authors:** Anna Canela-Xandri, Gemma Villorbina, Mercè Balcells, Xavier Fernández-Francos, Luisa F. Cabeza, Ramon Canela-Garayoa

**Affiliations:** 1 University of Lleida (UdL) and DBA Center, Av. Alcalde Rovira Roure, 191, 25198 Lleida, Spain; acanelxa@quimica.udl.cat (A.C.-X.); gemmav@quimica.udl.cat (G.V.); balcells@quimica.udl.cat (M.B.); 2 BarcelonaTech (UPC), Av. Diagonal 647, 08028 Barcelona; xavier.fernandez@upc.edu; 3 GREiA Research Group, INSPIRES Research Centre, Universitat de Lleida, Pere de Cabrera s/n, 25001 Lleida, Spain

**Keywords:** alkyl monoamides, glycerol, hydrogen bond, PCM, TES

## Abstract

Nine monoamides were synthesized from carboxylic acids (C8–C18) and crude glycerol. The final monoamides were the result of a rearrangement of the acyl chain during the final hydrogenation process. The purity of the final compounds was determined by spectroscopic and mass spectrometry (MS) techniques. The thermophysical properties of solid monoamides were investigated to determine their capability to act as phase change materials (PCM) in thermal energy storage. Thermophysical properties were determined with a differential scanning calorimeter (DSC). The melting temperatures of the analyzed material ranged from 62.2 °C to 116.4 °C. The analyzed enthalpy of these monoamides ranged from 25.8 kJ/kg to 149.7 kJ/kg. Enthalpy values are analyzed considering the carbon chain and the formation of hydrogen bonds.

## 1. Introduction

Phase change materials (PCM) for thermal energy storage (TES) are used in commercial applications such as ceiling and wall panels that can work day and night to stabilize indoor temperatures [1,2] and provide greater indoors thermal comfort. They have been implemented for cold storage [3], domestic hot water (DHW) [4], solar cooling [5], and concentrating solar solar power plants [6]. The PCM market can be segmented into three broad product categories: paraffin (45%), salt hydrates (33%), and biomass based PCM (biobPCM) (22%). There are already several commercial products involving these categories, and all of them have advantages and disadvantages, but their main feature is their heat storage capacity. PCMs with a heat storage capacity (∆H) greater than 100 kJ/kg are considered as suitable candidates for use as PCM. Typically, the products on the market have a ∆H of around 100 kJ/kg.

BiobPCM is one of the most efficient ways of storing thermal energy from abundant bio-based materials and has higher latent heat values than paraffin [1]. Many BiobPCM are based on fatty acids and fatty esters [7,8], although fatty diamides have also been studied for this purpose [9,10]. The strength and amount of hydrogen bonds have been indicated as key points in improving thermal properties of functionalized ionic liquids [11], and in polymeric phase change materials [12].

Hydrogen bonds (H-bonding) can be inter and intramolecular bonds. Their presence can be studied using FTIR, ^1^H NMR, and ^13^C NMR. The length of the X–H bond usually increases in the formation of the hydrogen bond, leading to a red shift of the infrared X–H stretching frequency and an increase in the infrared absorption cross-section for the X–H stretching vibration. The greater the lengthening of the X–H bond, the stronger the H ··· Y bond will be. The X–H ··· Y–Z hydrogen bond typically includes a pronounced proton unshielding for H in X–H [13]. The ^13^C NMR chemical shift is very sensitive to a changing charge density. The ^13^C NMR chemical shift represents the physicochemical property related to the specificity of the surrounding ^13^C atoms as a function of shielding or unshielding of their nuclei by electrons [14]. Factors such as backbone hydrogen bonding and electrostatic interactions also influence the shielding effect. Differential exposure to solvent is another characteristic with an established influence on chemical shifts [15]. H-bonding also has an influence on the increase of the melting points of compounds [16], although a simple correlation between the melting points and the hydrogen-bonding patterns is not always feasible [17]. For instance, a general increase in the melting point was observed with the increase in chain length as a function of molecular masses between 50 and 600 g/mol in saturated carboxylic esters of low-molecular weight alcohols. However, the melting points examined did not increase strictly linearly after increasing the molecular mass [7].

Recently, we described the synthesis of the IL 1,1′-(2-hydroxypropane-1,3-diyl)-bis(3-butylimidazol-1-ium) and 1,10-(2-(acryloyloxy)propane-1,3-diyl)-bis(3-butylimidazol-1-ium) salts from glycerol derivatives. We also obtained eutectic mixtures of fatty acids and alkyl esters of 9,10-didhydroxyestearic acid. In both cases, we described their thermal energy storage capacity [18,19,20].

**Figure 1:**
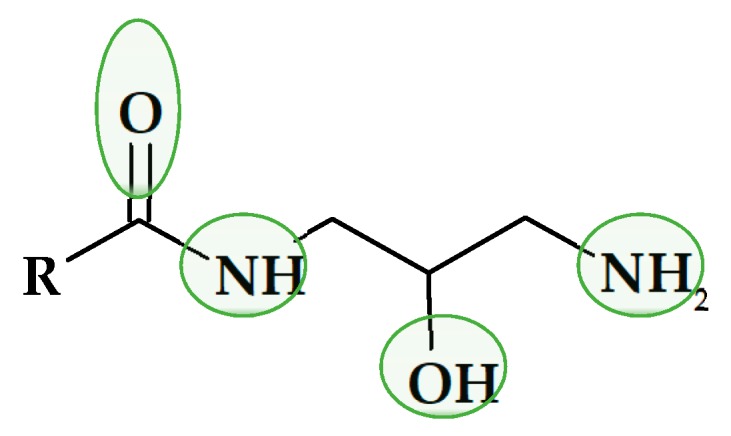
A likely hydrogen bond formation.

Joining both concepts, the preparation of biobPCMs based on carboxylic acids and glycerol, and the design of molecules with a high capacity of forming inter- and intra-molecular hydrogen bonds, led to the aim of the present study: to evaluate the thermal storage capacity of monoamides of 1,3-diamine-2-propanol. These compounds present four positions capable of forming hydrogen-bonds as donor or acceptors, as shown in Figure 1, which are powerful assembling tools due to their increased bonding strength, specificity, and directionality [21].

Here we report on the behavior of monoamides with a multicomponent H-bonding system that has three neighboring functional groups capable of forming at least four H-bonds. As part of the study, we modified the alkyl chain of the amide, maintaining the hydrophilic part of the molecule. Modification included: π-systems, alkyl chains of different lengths, and odd and even alkyl chains. Its thermal energy storage capacity and its fusion enthalpy were determined. We completed our study by using temperature-variable FTIR, which is a powerful tool for studing the presence of H-bonds [22]. High resolution nuclear magnetic resonance (NMR) spectroscopy, on the other hand, was also used as a powerful tool to obtain useful information on hydrogen bond formation [23].

## 2. Results and Discussions

**Figure 2:**
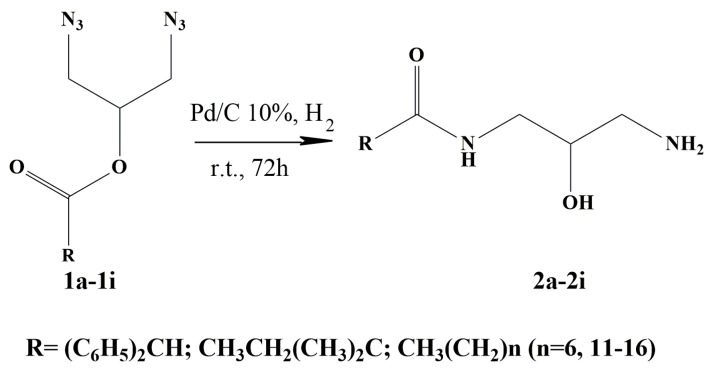
Scheme of the synthesis of monoamides **2a** to **2i** by hydrogenation of the corresponding diazides 1a to 1i.

Products **1a**–**1i** were synthesized using the method previously described by our group [24], using glycerol and the corresponding carboxylic acid as starting materials. Subsequently, the chloride was replaced by the azide group using a previously reported technique [25]. The azides were reduced by catalytic hydrogenation under mild conditions using Pd/C [26]. The reduction resulted in an O- to N-acyl migration to yield the expected monoamide (Figure 2). A similar rearrangement has been previously described [27]. Products **2b**–**2i** were obtained as solids with yields ranging 57% to 88%, while **2a** was a colorless oil (Table 1). Therefore, the thermal storage capacity of monoamides **2b**–**2i** was studied.

Table 1 shows the melting points of compounds **2b**–**2i**, many of which are anomalous compared to products of similar molecular weight, chemical structure, and expected polarity [28]. For compounds with an even number of carbons, these values are consistent with the presence of intermolecular H-bonds, which may confer extra stability to the interaction between individual molecules. Previous studies indicate that this increase in stability is reflected in an increase in the melting point [28]. For an odd number of compounds, erratic behavior can be observed. This may be due to the different packing capacity of the different compounds and their capacity to form inter and intramolecular H bonds [29].

Table 2 summarizes the results obtained by DSC, and includes the values of each property measured in the last two cycles of a sequence of three. Only those solid compounds that showed phase change transition under the described experimental conditions are indicated in the table. All DSC signals for the PCM under study are included in the Supporting Materials (Figures S1–S8). In these figures the enthalpy of phase change is presented as the normalized parameter obtained by integration of the area under each peak, while the phase change temperature is given by the peak temperature. Some solid monoamides (**2b**, **2c**, and **2f**) did not show a phase change transition under the described experimental conditions, as can be seen in the Supplementary Materials.

The melting temperatures of the analyzed materials ranged from 62.2 °C to 116.4 °C, while the solidification temperatures ranged from 57.8 °C to 105.7 °C. Monoamide **2i** had the largest difference between melting and solidification temperatures (21.9 °C), while **2d** had the smallest difference, (3.9 °C). The analyzed enthalpy of the monoamides ranged from 25.8 kJ/kg to 149.7 kJ/kg. The highest values of latent heat were of the same order as those of commercial PCMs with low values, such as paraffin wax (146–210 kJ/kg). This values were in the range of some diamides that have been described (103–148 kJ/kg), although they were lower than those of other diamides described (190 and 210 kJ/kg) [9]. The compounds **2e**, **2g**, and **2h** presented phase change enthalpies between 100 and 149 kJ/kg in their first cycle. However, these materials partially lost their latent heat storage capacity in the third analysis cycle; **2e** lost 8.3% of its melting enthalpy and 15.71% of its solidification enthalpy, while **2g** lost around the 27% and 12%, respectively.

In the light of the results, **2g** and **2h** presented the most suitable properties to be implemented as PCM. Monoamide **2h** showed the highest latent heat storage capacity (145.4 kJ/kg for melting and 129.3 kJ/kg for solidification for its second cycle, and 125.1 kJ/kg for melting and 122.0 kJ/kg for solidification for its third cycle). Monoamide **2g** presented the smallest difference between the solidification and melting temperatures (6% and 9% for the second and third cycle, respectively). Nevertheless, **2h** presented a significant decrease in its thermophysical properties after the second cycle.

After considering these results, we focused on the ^13^C NMR and FT-IR spectra of these compounds. ^1^H NMR was not considered because some of the monoamides were only soluble in methanol, which prevents seeing H-X bonds.

Table 3 shows the ∆ppm of the four carbons bound to atoms capable of participating in H-bonds and the alfa carbon of the acyl chain for monoamides **2d**, **2e**, **2g**, **2h**, and **2i**. ∆ppm was calculated for each compound with respect to the chemical shifts of the equivalen ^13^C in **2g**. Although α-C does not participate in traditional hydrogen bonds, its chemical shift is sensitive to the structure and residue identity [30].

The use of CD_3_OD instead of CDCl_3_ can lead to a slightly downfield shifts of the ^13^C bound to X···H atoms [31]. This behavior could explain the −∆ppm shown by the ^13^C of **2d** and **2i** indicated in the table. Similar behavior could be assigned to the ^13^COH in **2h** with a −1.5 ∆ppm, the highest ∆ppm observed for this type of carbon in all monoamides. However, they cannot explain the −∆ppm shown by **2e**, solubilized in CDCl_3_ as **2g**. Futhermore, ^13^C bound to the nitrogen in **2h** show a clear upfield shift not attributable to the solvent. These upfield shifts (over 11 ppm shift between **2h** and **2i** for ^13^CNH_2_) could result from the relative increase in electron density of these ^13^C, due to the formation of stronger H-bonds. This could indicate an increase in intramolecular H-bonds [30], which would contribute to the high ∆H_melting_ difference between **2h** and **2i**. The ^13^C shift of the carboxamide group was almost the same for all the compounds. This could indicate the same electronic behavior of the carbonyl of the five studied compounds, since the ^13^C NMR chemical shift is very sensitive to the change of charge density [14]. Finally, the downfield shifts showed for Cα in **2d** and **2i** could indicate clear differences in alkyl chain conformations between those compounds showing low ∆H_melting_ and those with ∆H_melting_ greater than 100 kJ/kg.

FTIR spectroscopy is a highly sensitive technique for detecting the formation of H-bonds [22]. The charge transfer between the proton acceptor (Y) and the proton donor (X-H) results in the elongation of the bond, which is reflected in a decrease in the X-H vibration frequency compared to that of a non-interacting specie.

The degree of intermolecular interaction of H-bonds depends on temperature and concentration. However, the compound **2i** was the only soluble in hot methanol. Consequently, FTIR studies were performed using the solid substances in a thermostatized ATR system. This system allowed us to record the FTIR from 40 °C to 135 °C.

A general overview of all the spectra recorded at 40 °C led us to consider the presence of H-bonds (see Supplementary Materials). Table 4 shows how an increase in temperature causes the band corresponding to the stretching vibration of O-H and N-H (from 3200 to 3500 cm^−1^) [32] to widen and shift to high wavenumber. This observation confirms the presence of intermolecular interactions, which weaken as the temperature increases. However, this band remains sharp and strong in the FTIR corresponding to monoamide **2h**. This spectrum was recorded at 135 °C, the highest temperature studied and clearly above the melting point of **2h**. If we focus on the region of the carbonyl stretching band, the temperature increase caused two clear effects: a decrease in the intensity of the band at 1634 cm^−1^, which corresponds to the participation of the C=O of the amide in the formation of H-bonds, and the emergence of a new bands beyond 1700 cm^−1^. An increase in temperature would decrease the interactions caused by the H-bonds. Consequently, the strength of the C=O bond increases, which is reflected in an increase in the wavenumber of the stretching vibration of C=O (amide I band) [32].

Again, this band remains sharp and strong in the FTIR spectrum recorded at 135 °C corresponding to monoamide **2h**. In contrast, the bands in the range 2917 to 2853 widen and lose intensity as the temperature increases, showing one increase of the entropy of the alkyl chain in all cases. The fact that the monoamide **2h** still maintains strong H-bonds beyond its melting point might support the hypothesis already indicated above of the presence of strong intramolecular H-bonds in this compound, which could justify its greater latent heat storage capacity compared to the other monoamides studied.

## 3. Materials and Methods

Starting materials and solvents were purchased from Sigma-Aldrich España (Madrid, Spain), Honeywell Fluka (Madrid, Spain), Supelco (Madrid, Spain) and Across(Madrid, Spain) and were used without further purification. The diazides (**1a**–**1i**) were synthesized from glycerol and the corresponding carboxylic acid following the procedure described before [24] (see Supplementary Materials). The crude product of the reaction was not purified due to its instability and was used for the hydrogenation reaction described below.

^1^H and ^13^C NMR were recorded on Varian AS400 MERCURYplus (^1^H, 400 MHz and ^13^C, 100 MHz, Agilent Technologies Spain, Las Rozas de Madrid, Spain), using CD_3_OD and CDCl_3_ as solvents. Spectra were recorded at 30 °C or 55 °C depending on the product solubility and using 20 sec of relaxation time. The chemical shifts (δ) are reported in ppm relative to the solvent used. Spin multiplicities are reported as a singlet (s), doublet (d), or triplet (t), with coupling constants (J) given in Hz, or multiplet (m).

The melting points were measured by open capillary tubes in a Gallenkamp apparatus (Impexron GMBH, Pfullingen, Germany). They are uncorrected.

A Bruker Vertex 70 FTIR spectrometer equipped with an attenuated total reflection (ATR) accessory (Golden GateTM, Specac Ltd., Bruker Española S.A., Rivas-Vaciamadrid, Spain) which is temperature-controlled (heated single-reflection diamond ATR crystal) was used to measure the FTIR spectra of the compounds at a range of temperatures. FTIR spectra were measured with a resolution of 4 cm^−1^ in a range between 600 and 4000 cm^−1^ and averaged over 20 scans. A correction for the dependence of absorbance on the wavelength in the ATR was performed. The infrared spectra of the samples were recorded at room temperature and then in a temperature interval between 40 °C and 125 °C. After reaching 125 °C, the samples were cooled to 40 °C and the spectra were recorded again in order to ensure product stability. We also recorded FTIR spectra with a Jasco FT/IR-6300 equipped with an ATR, in a range between 600 and 4000 cm^−1^.

High resolution mass spectra were recorded by direct infusion to a mass spectrometer Agilent G6510AA Q-TOF using ESI ionization.

The equipment used was a DSC 822e from Mettler Toledo (Mettler Toledo SA Española, L’Hospitalet de Llobregat, Spain). The methodology carried out to analyze the phase change enthalpy and temperature follows a three cycle program. The first cycle at 10 °C/min was disregarded and the mean value of the two following cycles was calculated. The second and the third cycles were performed at 0.5 °C/min heating and cooling rate under a nitrogen gas flow. The DSC analyses were performed between 45 °C and 125 °C. The amount of sample used was less than 5 mg and the sample was placed into 40 μI aluminum crucibles. Another sample of the materials which showed phase change enthalpies higher than 120 kJ/kg were analyzed under the same conditions. In addition, the equipment precision was ±0.1 °C for temperature and ±3 kJ/kg for enthalpy measurements.

### 3.1. General Procedure for the Synthesis of N-(3-amino-2-hydroxypropyl)amides **2a** to **2i**

10% Pd/C (10 wt %) suspended in MeOH (1.0 mL) was added to a stirred solution of the diazide (**1a**–**1i**) (1.0 mmol) in MeOH (1 mL). The system was sealed with a septum and vigorously stirred at 25 °C after two vacuum/H_2_ cycles. The reaction was conducted for 72 h under positive hydrogen pressure using a H_2_ balloon connected to the septum. The crude product of reaction was filtrate using a polyethylene frit and a celite bed. The solution was dried under vacuum to yield a crude white powder for all compounds, except 2a, which was a colourless oil. The solids were further purified by crystallization, using hot methanol as solvent for **2b**, **2c**, **2h** and **2i**, and AcOEt:MeOH (90:10) for the others. Compound **2a** was not further purified, as it was considered to be pure enough once extracted because of its ^1^H NMR spectra.

*N-(3-Amino-2-hydroxypropyl)-2,2-dimethylbutanamide* (**2a**) was obtained as a colourless oil. ^1^H NMR (400 MHz, CDCl_3_) δ 6.14 (1 H, s), 3.55 (1 H, m), 3.46–3.36 (1H, dd, *J* = 25.7; 9.4), 3.20–3.12 (1H, dddd, *J* = 20.3; 4.8), 2.75–2.73 (1H, dd, *J* = 19.9; 5.8), 2.58–2.51 (1H, m), 1.48 (2H, q), 1.10 (6H, m), 0.78 (3H, t). ^13^C NMR (101 MHz, CDCl_3_) δ 178.95, 71.18, 44.68, 43.16, 42.45, 33.82, 24.96, 24.94, 9.20. IR (ATR/ν) 3342, 2966, 2933, 2874, 1632, 1530, 1475, 1460, 1364, 1266, 1197, 1089, 1049, 945, 754, 665 cm^−1^. HRMS (ESI) *m/z* calcd. for C_9_H_21_N_2_O_2_ [M + H]^+^ 189.1598, found 189.1598.

*N-(3-Amino-2-hydroxypropyl)-2,2-diphenylacetamide* (**2b**) was obtained as a white power. ^1^H NMR (400 MHz, CD_3_OD) δ 7.37–7.21 (10 H, m), 6.10 (1H, s), 3.7 (1H, m), 3.57–3.46 (1H, dd, *J* = 13.7; 6.9), 3.36–3.26 (1H, dd, *J* = 11.9; 4.1), 2.86 (1H, dd, *J* = 13.9; 6.4), 2.67 (1H, dd, *J* = 11.9, 3.9). ^13^C NMR (101 MHz, CDCl_3_) δ 172.74, 139.33, 128.81, 128.68, 127.20, 77.22, 76.91, 76.59, 70.93, 59.23, 44.69, 43.51. IR (ATR/ν) 3315, 3065, 2924, 2859, 1644, 1582, 1532, 1495, 1449, 1368, 1252, 1175, 1031, 695 cm^−1^. HRMS (ESI) *m/z* calcd for C_17_H_21_N_2_O_2_ [M + H]^+^ 285.1598, found 285.1600.

*N-(3-Amino-2-hydroxypropyl)octanamide* (**2c**) was obtained as a white solid. ^1^H NMR (400 MHz, CD_3_OD) δ 3.67 (1H, m), 3.28–3.18 (2H, m), 2.76–2.72 (1H, dd, *J* = 13.1; 4.1), 2.63–2.57 (1H, dd, *J* = 13.2; 7.5), 2.20 (2H, t), 1.60 (2H, t), 1.31 (8H, m), 0.90 (3H, t, *J* = 6.9).^13^C NMR (101 MHz, CD_3_OD) δ 175.51, 69.81, 43.55, 42.45, 35.60, 31.49, 28.92, 28.68, 25.55, 22.25, 12.99. IR (ATR/ν): 3354; 3302; 2916; 2845;16392; 1549.; 1470 cm^−1^. HRMS (ESI) *m/z* calcd. for C_11_H_25_N_2_O_2_ [M + H]^+^ 217.1911, found 217.1912.

*N-(3-Amino-2-hydroxypropyl)tridecanamide* (**2d**) was obtained as a white solid. ^1^H NMR (400 MHz, CD_3_OD) δ 3.63 (1 H, ddd, *J* = 11.6; 8.0; 5.1), 3.28–3.16 (2H, tt, *J* = 11.1; 6.7), 2.70–2.64 (1H, dd, *J* = 20.4; 5.7), 2.60–2.51 (1H, dd, *J* = 13.2; 5.5), 2.20 (2H, t), 1.60 (2H, t), 1.29 (18H, m), 0.90 (3H, t, *J* = 6.8). ^13^C NMR (101 MHz, CD_3_OD) δ 175.35, 70.52, 43.76, 42.42, 35.11, 31.33, 29.21, 25.42, 22.28, 12.92. IR (ATR/ν): 3338; 3299; 2920; 2917; 2870; 2850; 1642; 1615; 1550; 1471 cm^−1^. HRMS (ESI) *m/z* calcd. for C_16_H_35_N_2_O_2_ [M + H]^+^ 287.2693, found 287.2687.

*N-(3-Amino-2-hydroxypropyl)tetradecanamide* (**2e**) was obtained as a white solid. ^1^H NMR (400 MHz, CDCl_3_) δ 5.91 (s, 1H), 3.54 (m, 1H), 3.46–3.38 (1H, ddd, *J* = 14.4; 6.4; 3.17–3.08 (1H, m), 2.81–2.71 (1H, dd, *J* = 12.7; 4.1), 2.57–2.52 (1H, dd, *J* = 12.7, 7.4), 2.13 (2H, t, *J* = 7.7), 1.56 (2H, m), 1.18 (19H, s), 0.88 (3H, t, *J* = 6.9). ^13^C NMR (101 MHz, CD_3_OD) δ 175.35, 70.64, 44.16, 42.39, 35.63, 31.65, 29.35, 29.33, 29.21, 29.05, 28.93, 25.61, 22.31, 13.01. IR (ATR/ν): 3356; 3083; 2966; 2955; 2872; 1593; 1402 cm^−1^. HRMS (ESI) *m/z* calcd. for C_17_H_37_N_2_O_2_ [M + H]^+^ 301.2850, found 301.2876.

*N-(3-Amino-2-hydroxypropyl)pentadecanamide* (**2f**) was obtained as a white solid. ^1^H NMR (400 MHz, CDCl_3_) δ 5.90 (1H, s), 3.55 (1 H, td, *J* = 7.2; 3.7), 3.45–3.05 (2H, m), 2.65 (2H, ddd, *J* = 20.1; 12.7; 5.8), 2.13 (2H, t, *J* = 7.7), 1.61–1.49 (2H, t), 1.20 (20H, m), 0.81 (3H, t, *J* = 6.9). ^13^C NMR (101 MHz, CDCl_3_) δ 174.14, 70.97, 44.56, 43.04, 36.74, 31.91, 29.66, 29.63, 29.60, 29.47, 29.34, 29.29, 25.74, 22.68, 14.12. IR (ATR/ν): 3310; 2956; 2915; 2870; 2849; 1646; 1607; 1540; 1470cm^−1^. HRMS (ESI) *m/z* calcd. for C_18_H_39_N_2_O_2_ [M + H]^+^ 315.3006, found 315.3004.

*N-(3-Amino-2-hydroxypropyl)hexadecanamide* (**2g**) was obtained as a white solid. ^1^H NMR (400 MHz, CDCl_3_) δ 5.90 (1H, s), 3.55 (1H, ddd, *J* = 11.1; 7.4; 4.10), 3.45–3.38 (1 H, ddd, *J* = 11.3; 7.2; 3.7), 3.17–3.07 (1H, m), 2.82–2.71 (1H, dd, *J* = 12.7; 5.8), 2.57–2.49 (1H, dd, *J* = 12.2; 7.7), 2.13 (2H, m), 1.16 (2H, m), 1.18 (24H, s), 0.81 (3 H, t, *J* = 6.9). ^13^C NMR (101 MHz, CD_3_OD) δ 175.39, 70.81, 44.23, 42.39, 35.64, 31.65, 29.36, 29.33, 29.31, 29.20, 29.05, 28.92, 25.61, 22.31, 13.02. IR (ATR/ν): 3360; 3299; 2920; 2916; 2872; 2853; 1639; 1593; 1551; 1471; 1461 cm^−1^. HRMS (ESI) *m/z* calcd. for C_19_H_41_N_2_O_2_ [M + H]^+^ 329.3163, found 329.3165.

*N-(3-Amino-2-hydroxypropyl)heptadecanamide* (**2h**) was obtained as a white solid. ^1^H NMR (400 MHz, CD_3_OD) δ 3.70 (1H, m), 3.27–3.18 (2 H, m), 2.80–2.76 (1H, ddd, *J* = 13.1; 4.0), 2.66–2.61 (1H, m), 2.21 (2H, dd, *J* = 14.6; 7.8), 1.60 (2H, m), 1.28 (26H, s), 0.89 (3H, t, *J* = 6.8). ^13^C NMR (101 MHz, CD_3_OD) δ 175.52, 69.28, 48.20, 47.99, 47.85, 47.78, 47.64, 47.57, 47.39, 47.35, 47.14, 46.93, 43.53, 42.42, 35.60, 31.64, 29.35, 29.20, 29.04, 28.94, 25.57, 22.30, 13.00. IR (ATR/ν):3360; 3299, 2920; 2853; 1639; 1527; 1495; 1456 cm^−1^. HRMS (ESI) *m/z* calcd. for C_20_H_43_N_2_O_2_ [M + H]^+^ 343.3319, found 343.3318.

*N-(3-Amino-2-hydroxypropyl)octadecanamide* (**2i**) was obtained as a white solid. ^1^H NMR (400 MHz, CD_3_OD) δ 3.64 (1H, m), 3.26–3.17 (2 H, m), 2.73–2.65 (1 H, dd, *J* = 20.3; 5.7), 2.60–2.53 (1 H, dd, *J* = 13.2; 6.2), 2.20 (2 H, t, *J* = 14.8; 7.0), 1.60 (2H, m), 1.28 (28H, d, *J* = 14.0), 0.89 (3H, t, *J* = 6.7). ^13^C NMR (101 MHz, CD_3_OD) δ 175.35, 70.53, 42.53, 35.68, 31.56, 29.26, 29.12, 28.94, 25.52, 22.21, 12.89. IR (ATR/ν): 3357; 3303; 2917; 2853; 1642; 1556; 1469 cm^−1^. HRMS (ESI) *m/z* calcd. for C_21_H_45_N_2_O_2_ [M + H]^+^ 357.3476, found 357.3472.

### 3.2. Thermoanalytical Methods

The sample mass used was around 15 mg using 100 µL aluminum crucibles under a N_2_ flow of 80 mL/min. A dynamic mode was used taking the melting temperature (Tm) of the sample into account. This method is based on a 0.5 K/min heating constant rate from 10 degrees below the Tm of material to 10 degrees above it.

Evaluation of the resulting DSC curves was performed with STARe v.11.00 software from Mettler-Toledo. Phase change enthalpy and temperature were obtained from the DSC heat flux signal response by integration. Specifically, for phase change temperature, the peak temperature was considered as the representative temperature of a phase change material.

## 4. Conclusions

A new set of monoamides of 1,3-diamine-2-propanol has been synthesized from crude glycerol and various carboxylic acids. These compounds can form at least 4 hydrogen-bonds, which are powerful assembling tools. Monoamides were investigated considering their thermophysical properties to be used as phase change materials (PCM) in thermal energy storage. The thermophysical properties were determined with a differential scanning calorimeter (DSC). In light of the results, compounds **2g** and **2h** presented the most suitable properties to be implemented as PCM. In addtion, we demonstrated the influence of H-bonds and the alkyl chain on these properties. In this regard, the relevance of the inter and intramolecular interactions of these molecules has been reinforced by the study of FT-IR at low temperature and temperatures beyond the melting points and the ^13^C NMR shifts

When working with a thermal energy storage (TES) system, it is critical to ensure a long term performance. The stability of the materials under real working conditions will guarantee the efficiency of the system and determine its life time. An essential parameter to be considered is thermal cycling stability, which is the capacity of a material to keep its thermal properties almost constant during a specific number of melting/freezing thermal cycles. A thermal cycling stability study allows evaluating the durability of a PCM for a certain application. In this study, three cycles were performed for each sample, the first cycle at 10 °C/min and two analysis cycles, and a significant (10–30%) drop of the material phase change enthalpies was observed. In addition, we are currently performing thermal cycling stability tests (10, 100 and 1000 cycles, for example) with monoamides **2g** and **2h** to properly characterize the changes in the thermophysical and chemical properties of likely PCM during thermal cycling.

## Figures and Tables

**Table 1:** Summary of the physico-chemical properties of the products synthesized.

	R	Yield (%) ^a^	Melting Point (°C)
**2a**	CH_3_CH_2_C(CH_3_)_2_	93.1	–
**2b**	(C_6_H_5_)_2_CH	59.9	99.7–103.8
**2c**	CH_3_(CH_2_)_6_	68.2	114.8–119.0
**2d**	CH_3_(CH_2_)_11_	62.9	62.1–64.3
**2e**	CH_3_(CH_2_)_12_	56.6	112.9–115.5
**2f**	CH_3_(CH_2_)_13_	88.3	115.0–117.4
**2g**	CH_3_(CH_2_)_14_	74.7	112.1–114.9
**2h**	CH_3_(CH_2_)_15_	85.7	97.1–98.5
**2i**	CH_3_(CH_2_)_16_	69.1	110.9–113.1

^a^ Products were purified by silica gel column chromatography.

**Table 2:** Summary of the differential scanning calorimeter (DSC)results of the solid monoamides showing phase change transition under the experimental conditions assayed.

	R	Cycle	∆H_melting_ (kJ/kg)	∆H_solidification_ (kJ/kg)	T_melting_ (°C)	T_solidification_ (°C)
**2d**	CH_3_(CH_2_)_11_	2	25.8	26.0	62.2	58.0
		3	31.3	27.8	61.7	57.8
**2e**	CH_3_(CH_2_)_12_	2	107.2	101.8	116.4	101.0
		3	98.3	85.8	112.1	95.9
**2g**	CH_3_(CH_2_)_14_	2	149.7	118.1	111.5	105.7
		3	107.3	103.9	113.9	103.9
**2h**	CH_3_(CH_2_)_15_	2	145.4	129.3	96.6	85.3
		3	125.1	122.0	95.2	82.2
**2i**	CH_3_(CH_2_)_16_	2	67.3	96.0	111.0	90.8
		3	76.0	96.5	109.8	87.9

**Table 3:** ∆ppm taken with respect to the ^13^C chemical shifts of **2g** of the four carbons bound to atoms able to participate in H-bonds and the α-carbon of the acyl chain.

	R	Cα	C=O	CNH	COH	CNH_2_	Solvent	[Sample] (M)
**2d**	CH_3_(CH_2_)_11_	−0.5	0	−0.5	−0.3	0	CD_3_OD	0.07
**2e**	CH_3_(CH_2_)_12_	0	0	0	−0.2	0	CDCl_3_	0.07
**2g**	CH_3_(CH_2_)_14_	35.6	175.4	44.2	70.8	42.4	CDCl_3_	0.06
**2h**	CH_3_(CH_2_)_15_	0	0.1	4	−1.5	4.7	CD_3_OD	0.06
**2i**	CH_3_(CH_2_)_16_	−4	0	−1.7	−0.3	−6.7	CD_3_OD	0.06

**Table 4:** Effect of temperature in the FTIR signals of characteristics bond vibration in some of the monoamides synthesized.

	R	T (°C)	NH/OH (cm^−1^)	CH_2_ (cm^−1^)	CON (cm^−1^)
**2d**	CH_3_(CH_2_)_11_	30	3338 (w)/3299 (s)	2920 (s)/2850 (s)	1642 (s)
		125	3319 (m/b)	2917 (m)/2853 (m)	1729 (w)/1703 (w)/1646 (m)
**2g**	CH_3_(CH_2_)_14_	30	3360 (w)/3299 (s)	2920 (s)/2853 (s)	1639 (s)
		125	3321 (m/b)	2920 (m)/2853 (m)	1732 (w)/1703 (w)/1639 (w)
**2h**	CH_3_(CH_2_)_15_	30	3360 (w)/3299 (s)	2917 (s)/2853 (s)	1649 (s)
		135	3350 (w/b)/3309 (s)	2917(s)/2853 (s)	1729 (w)/1649 (s)
**2i**	CH_3_(CH_2_)_16_	30	3357 (w)/3303 (s)	2917 (s)/2853 (s)	1642 (s)
		125	3305 (m/b)	2917 (m)/2853 (m)	1729 (w)/1703 (w)/1646 (w)

w = weak band; m = medium band; s = strong band; /b = broad peak.

## Supplementary Materials

The following are available online. General scheme (synthetic process) pag S2, Thermophysical charaxterization (DSC response) pag S3 FT-IR spectra pag S7.
