# Supplementary material for: Synthesis and Thermophysical Characterization of Fatty Amides for Thermal Energy Storage
Source: Molecules. 2019 Oct 21;24(20):3777. doi: 10.3390/molecules24203777 (PMC6832967; doi:10.3390/molecules24203777)

SUPPLEMENTARY MATERIAL

SYNTHESIS AND THERMOPHYSICAL CHARACTERIZATION OF FATTY AMIDES FOR THERMAL ENERGY STORAGE

INDEX

GENERAL SCHEME (SYNTHETIC PROCESS) S2

THERMOPHYSICAL CHARAXTERIZATION (DSC response) S3

FT-IR SPECTRA S11

NMR SPECTRA S13

GENERAL SCHEME (SYNTHETIC PROCESS)

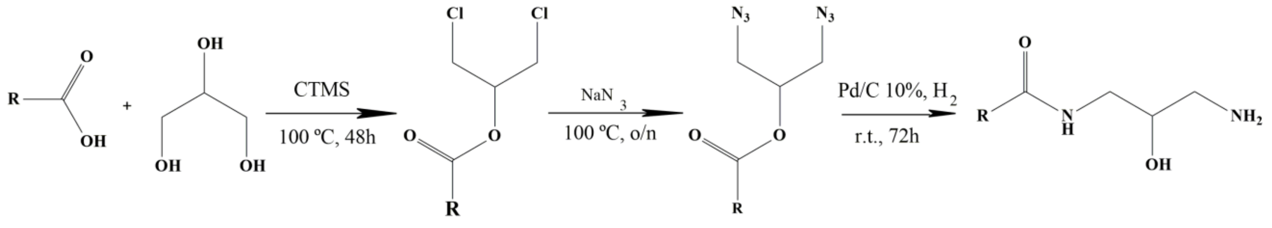

R= $(C_6H_5)_2CH$; $CH_3CH_2(CH_3)_2C$; $CH_3(CH_2)n$ (n=6, 11-16)

THERMOPHYSICAL CHARAXTERIZATION (DSC response)

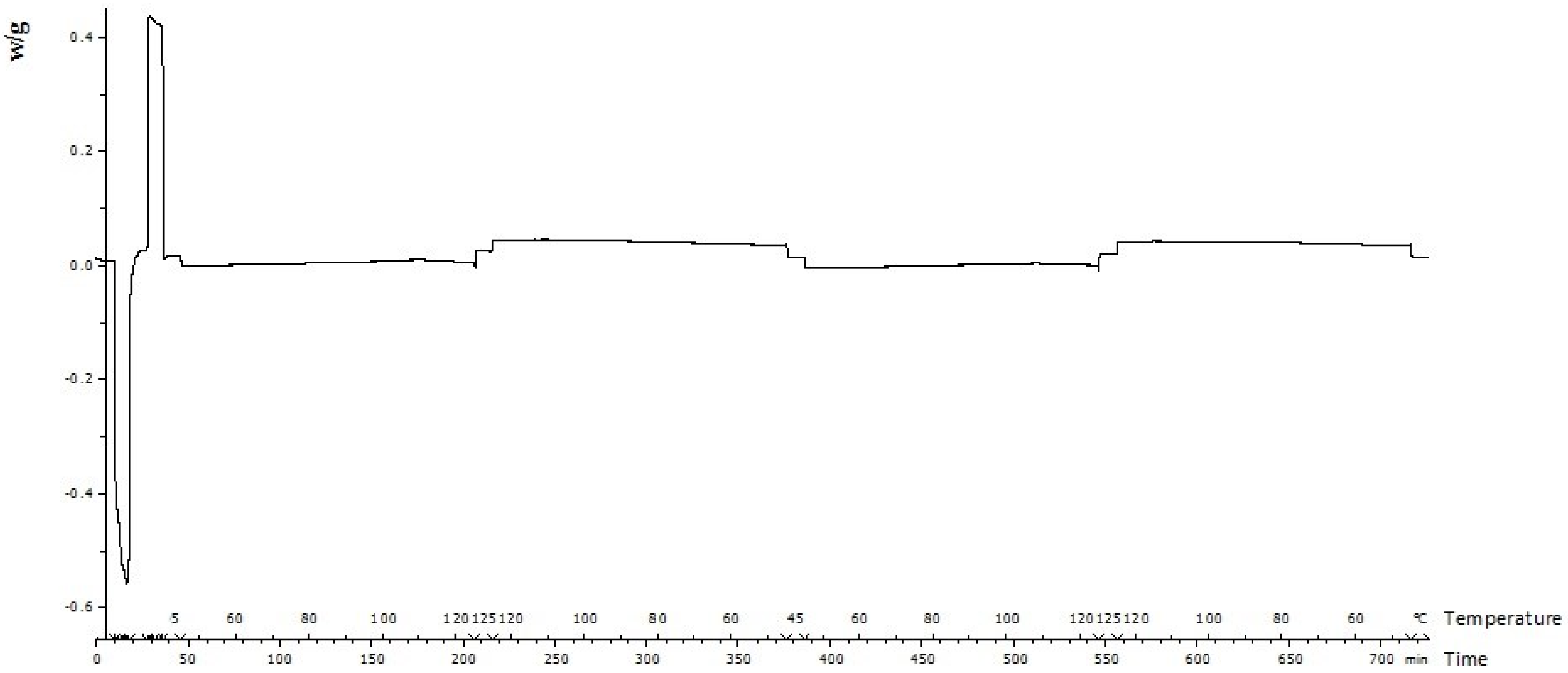

Figure 1. DSC response and evaluation of monoamide **2b**.

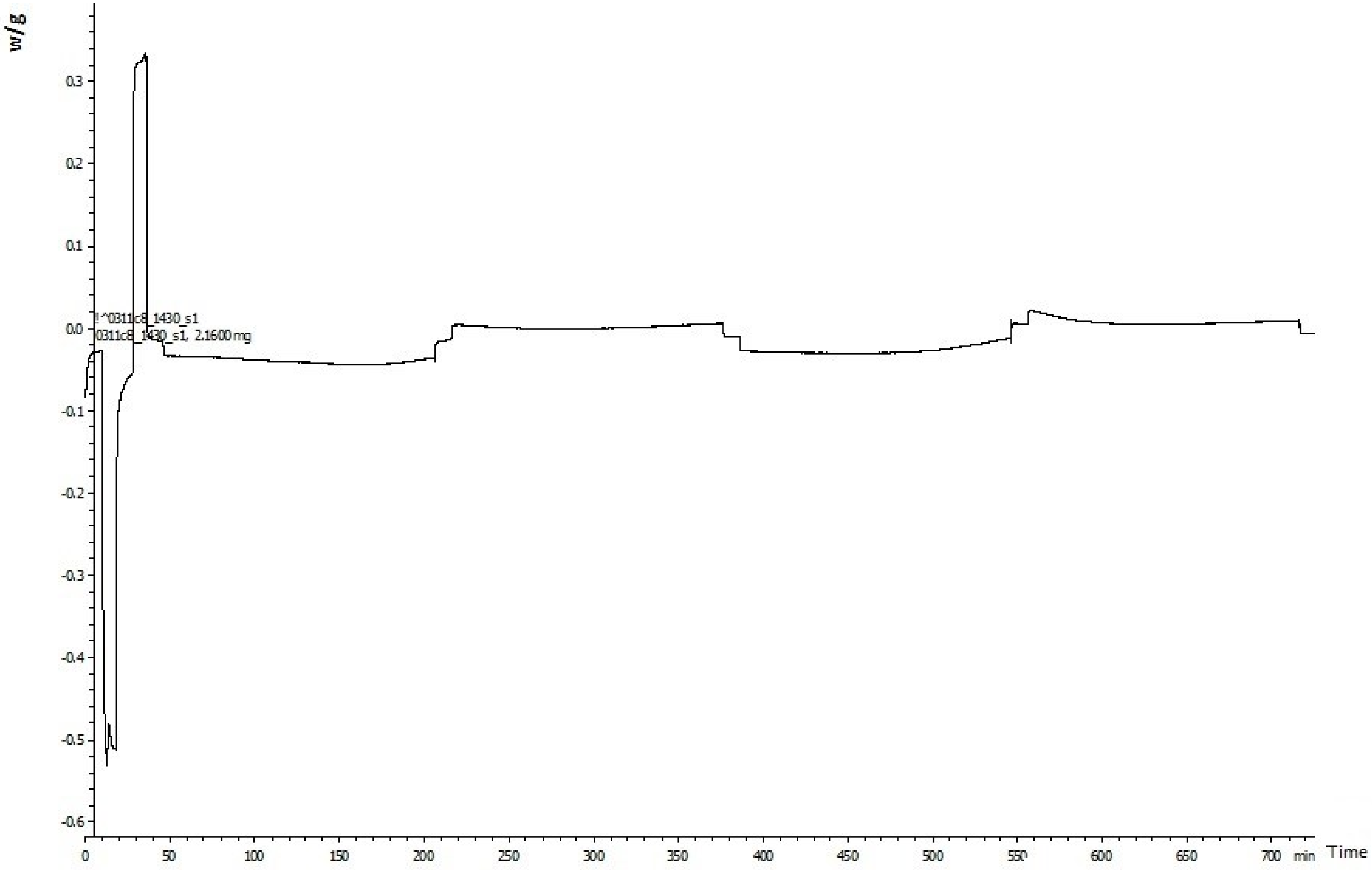

Figure 2. DSC response and evaluation of monoamide **2c**.

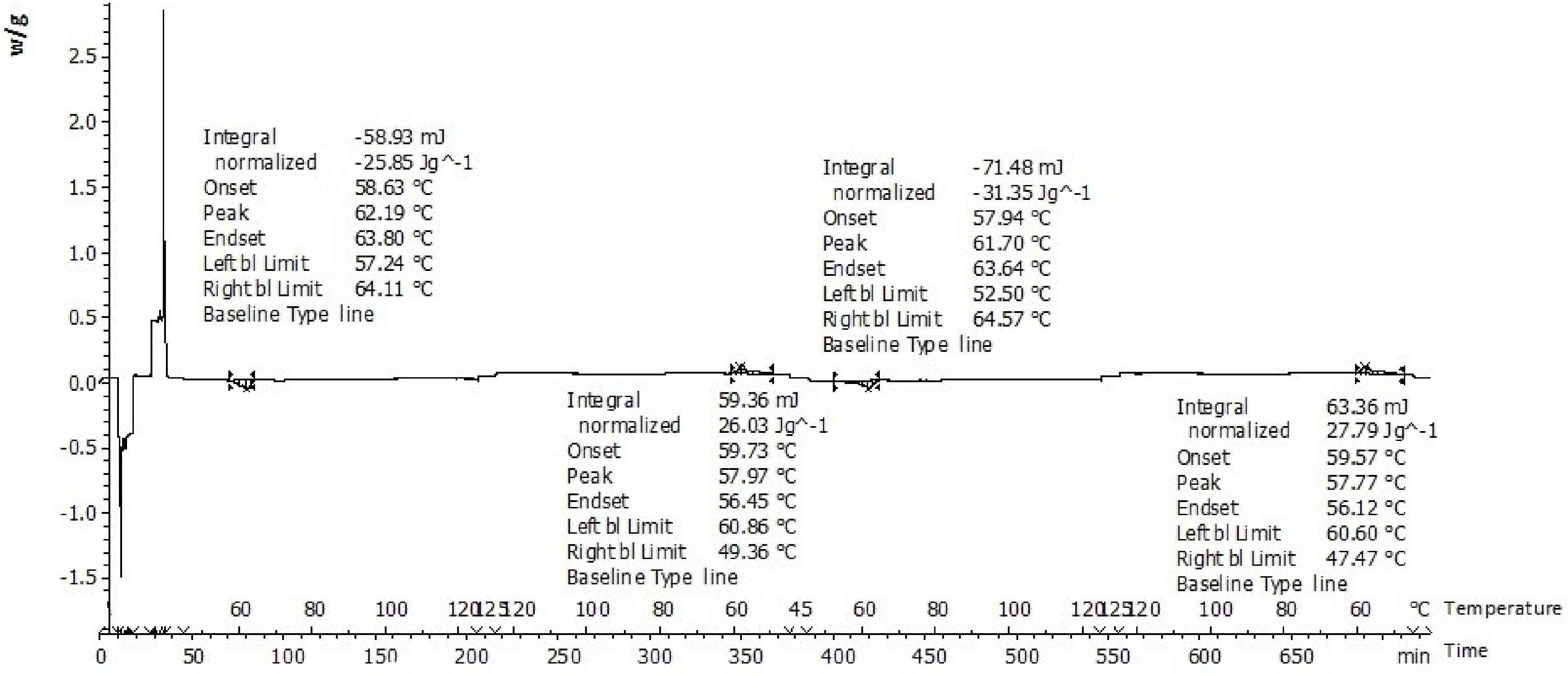

Figure 3. DSC response and evaluation of monoamide **2d**.

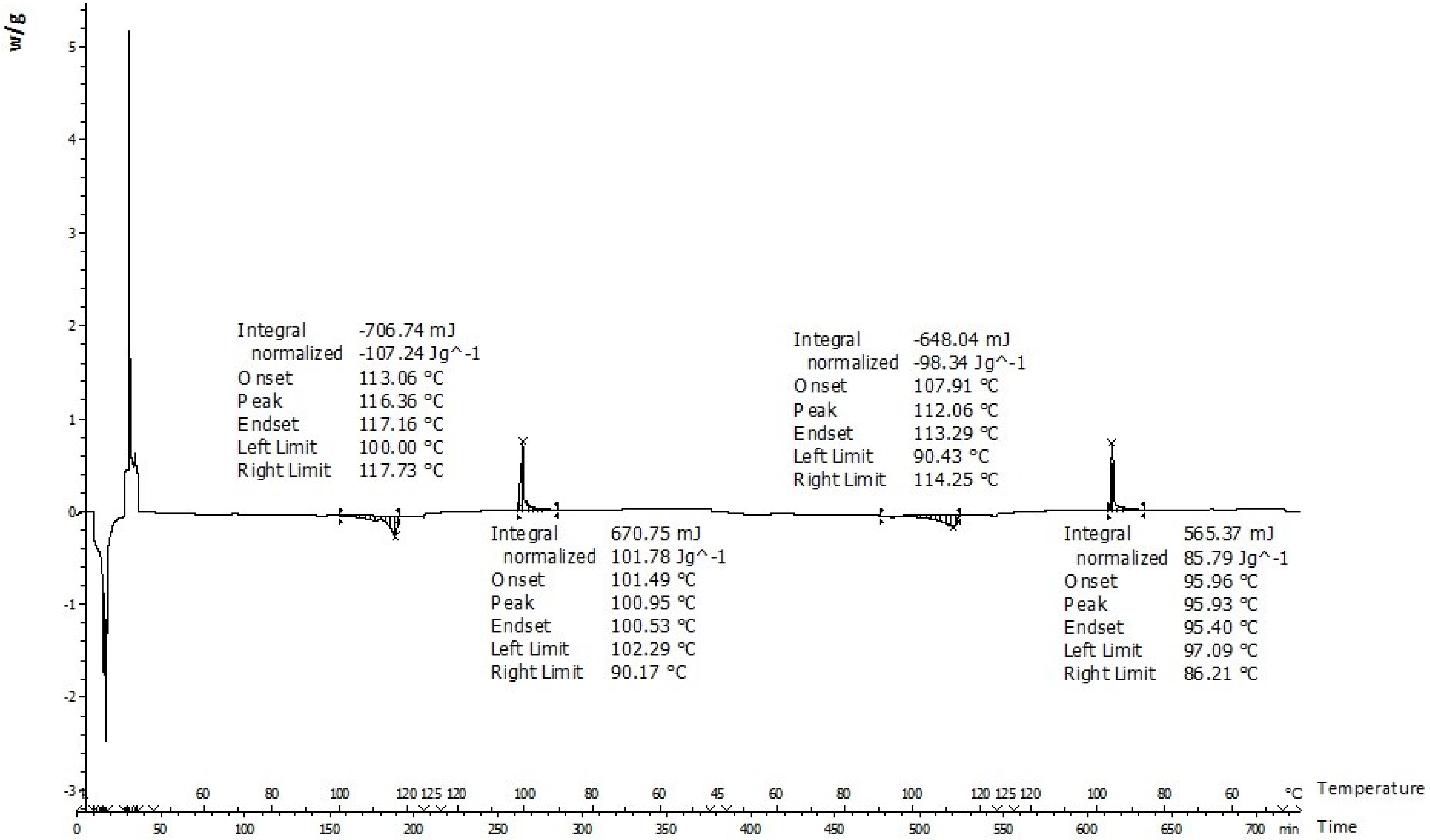

Figure 4. DSC response and evaluation of monoamide **2e**.

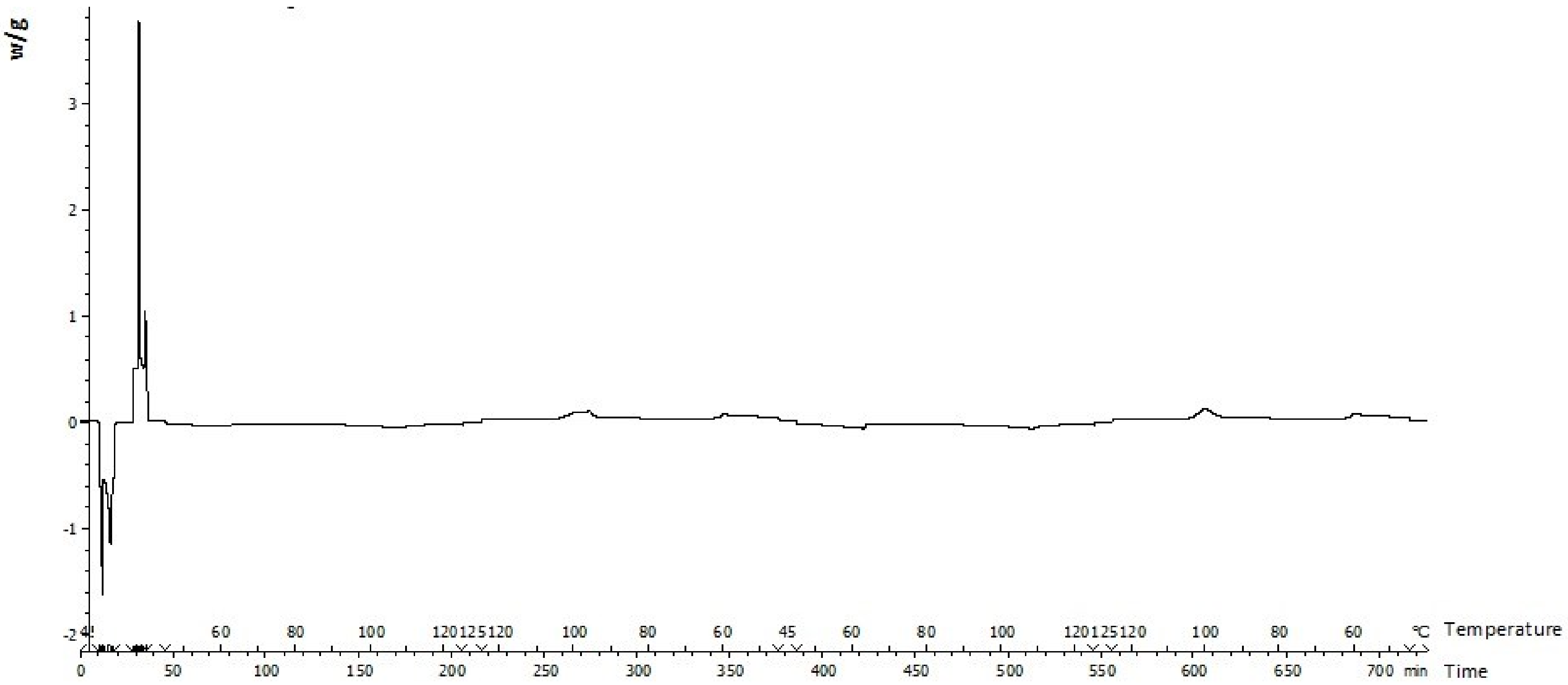

Figure 5. DSC response and evaluation of monoamide **2f**.

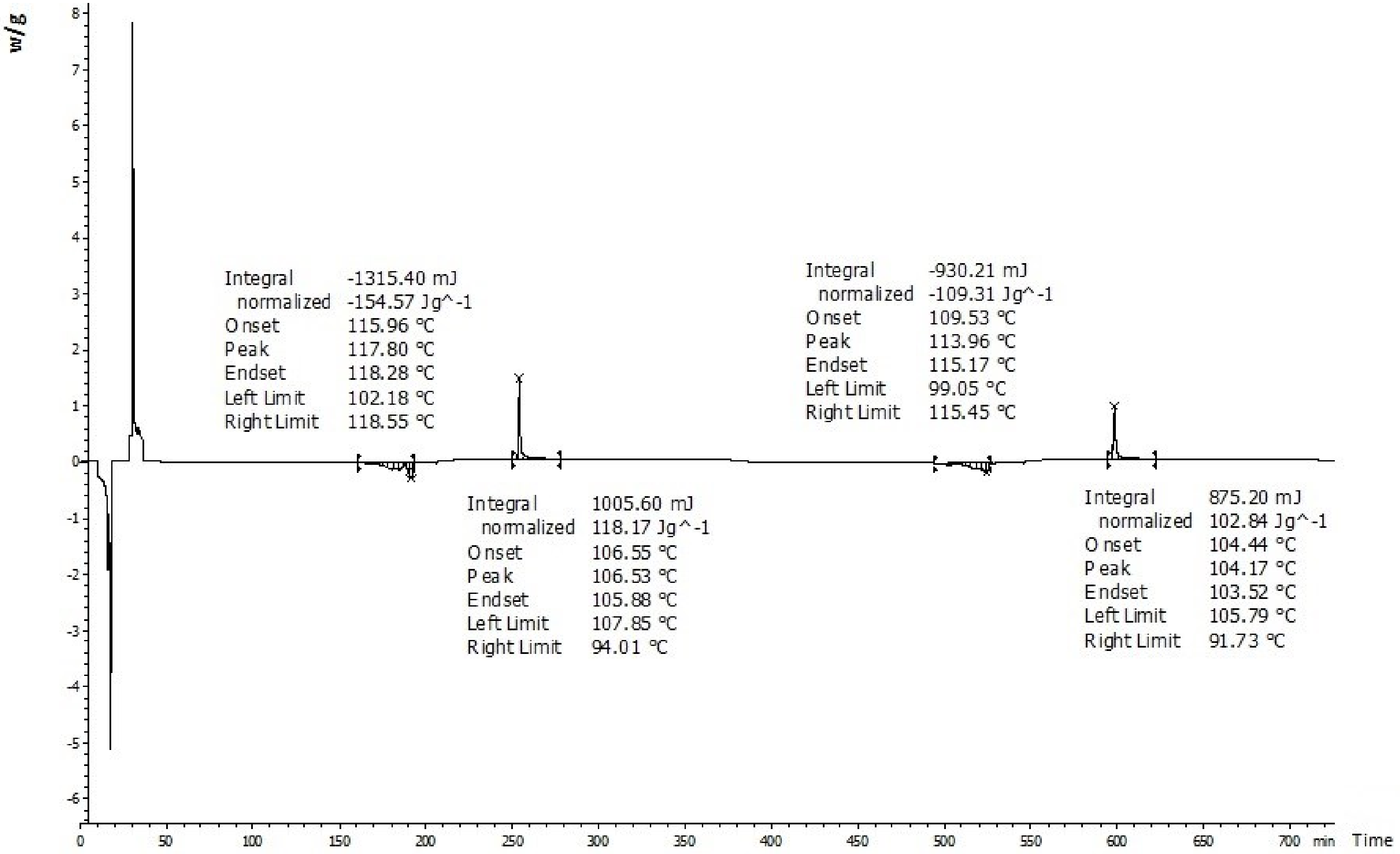

Figure 6. DSC response and evaluation of monoamide **2g**

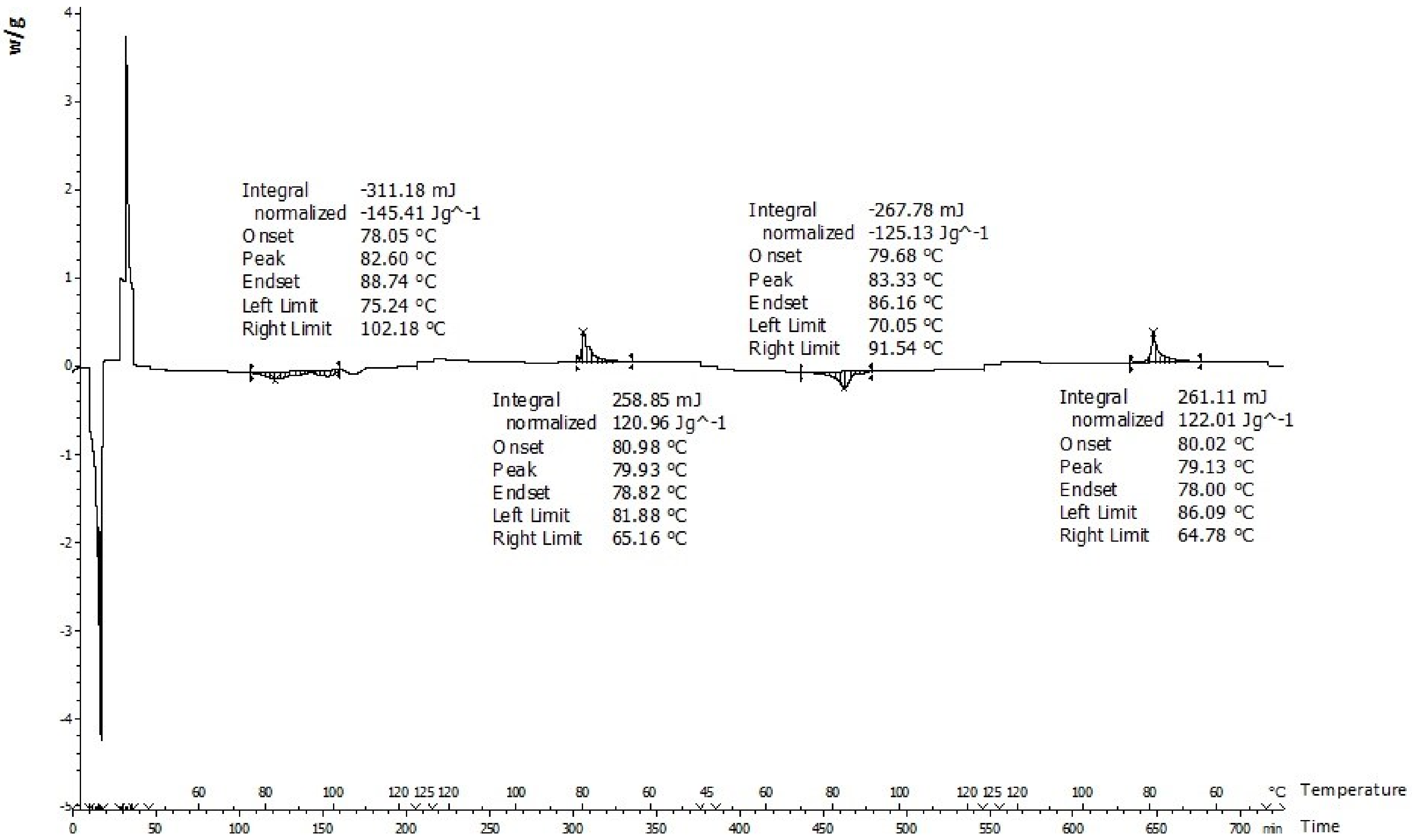

Figure 7. DSC response and evaluation of monoamide **2h**.

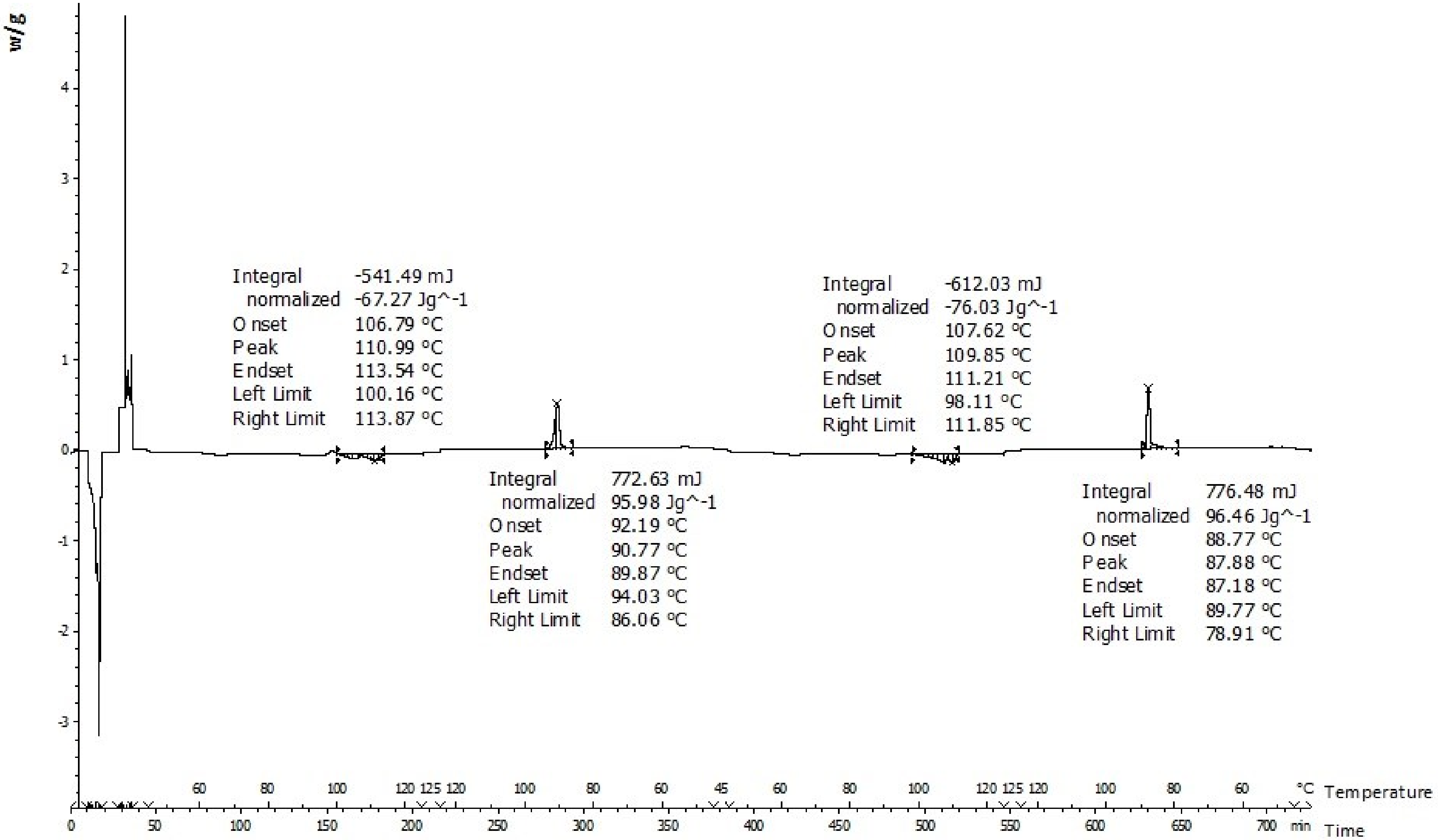

Figure 8. DSC response and evaluation of monoamide **2i**.

FT-IR SPECTRA

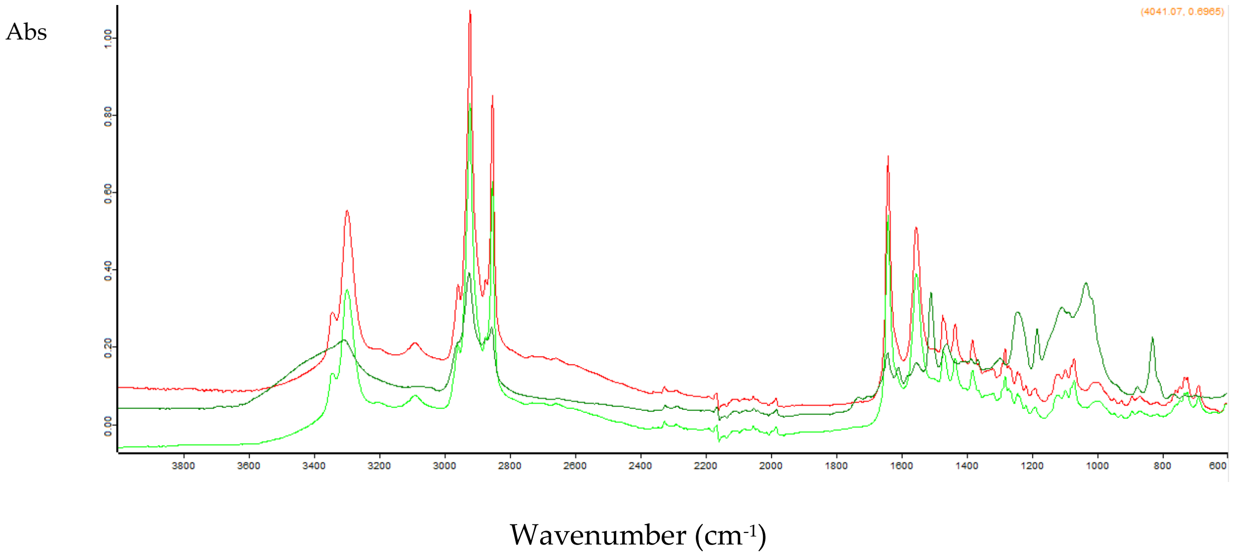

Figure 9. FT-IR spectrum of at 40ºC, 125ºC and 40ºC of monoamide **2d (**see color code at the bottom of the last page**)**.

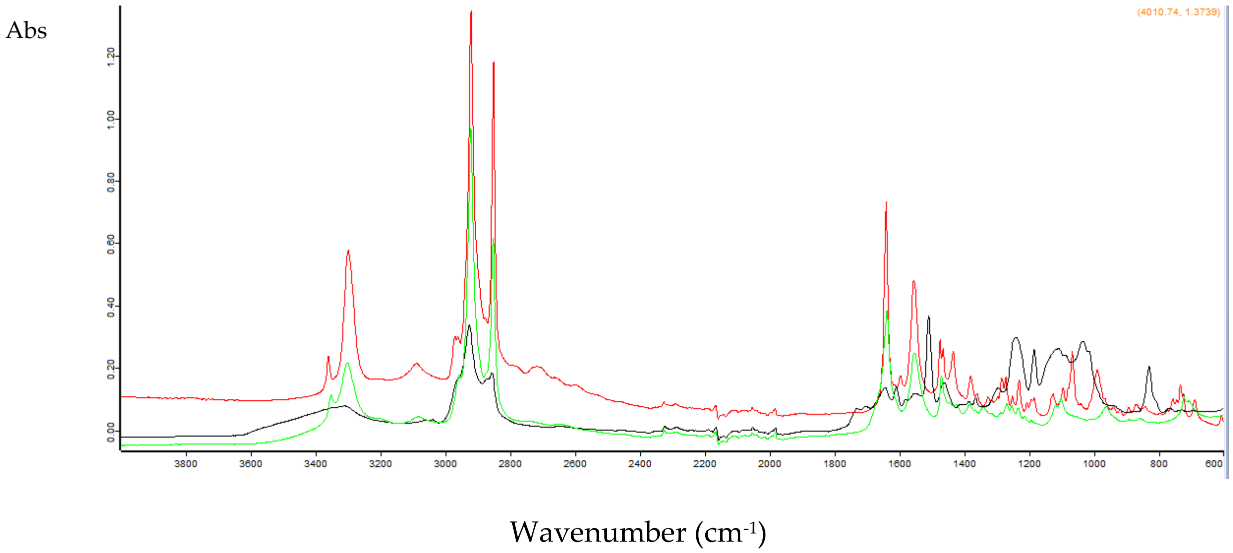

Figure 9. FT-IR spectrum of at 40ºC, 125ºC and 40ºC of monoamide **2g (**see color code at the bottom of the last page**)**.

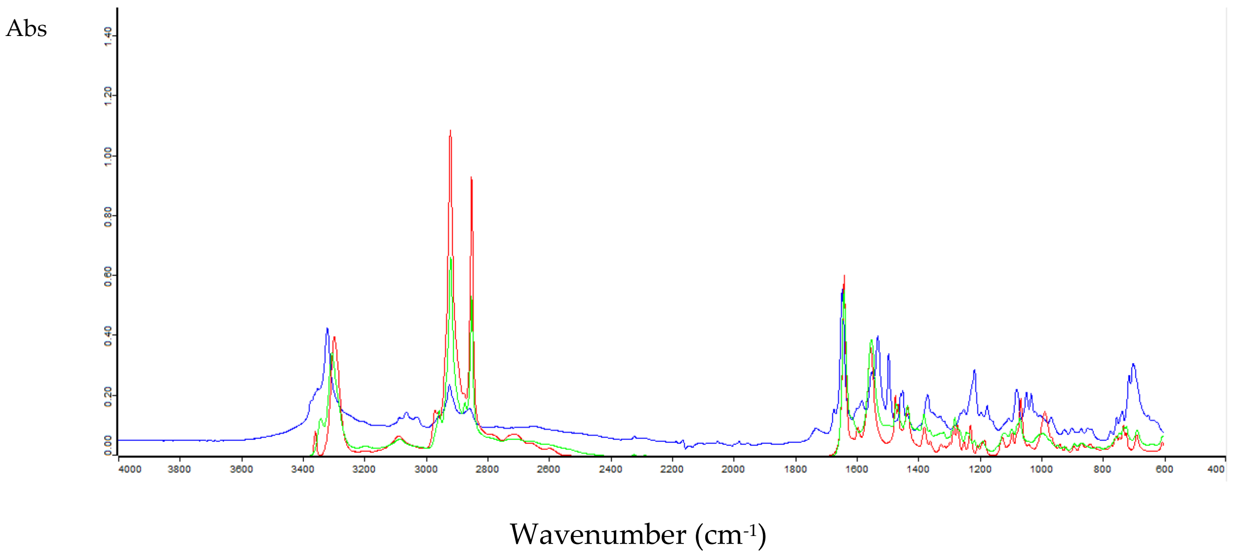

Figure 9. FT-IR spectrum of at 40°C, 135°C and 40°C of monoamide **2h (**see color code at the bottom of the last page**)**.

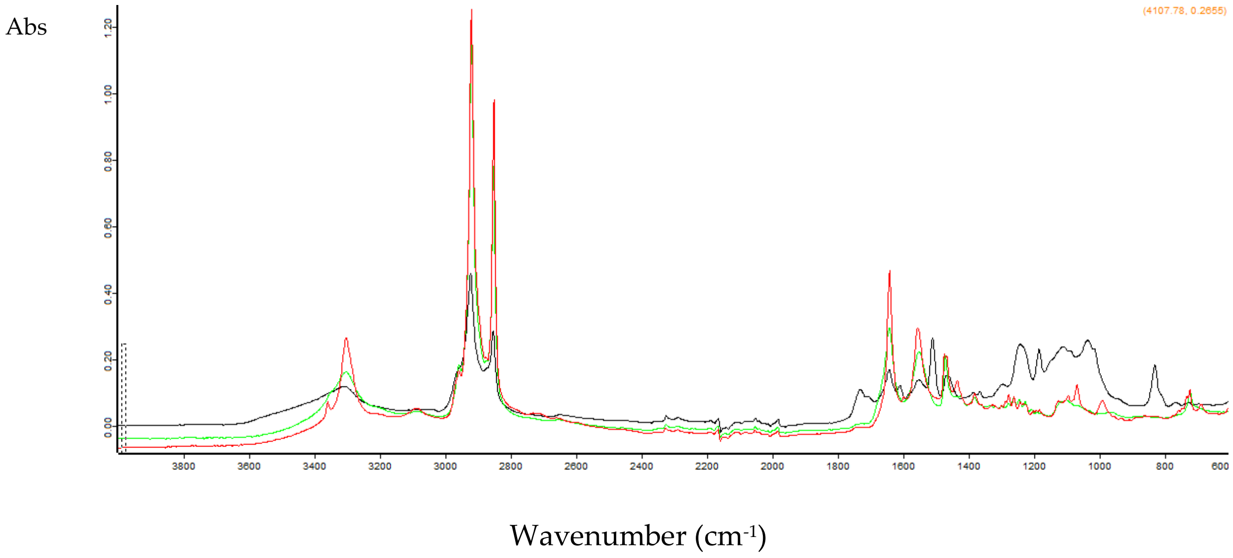

Figure 9. FT-IR spectrum of at 40°C, 125°C and 40°C of monoamide **2i (**see color code at the bottom of the last page**)**.

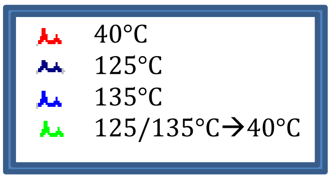

NMR SPECTRA

2d ^{1}H NMR (400 MHz, CD_3OD)

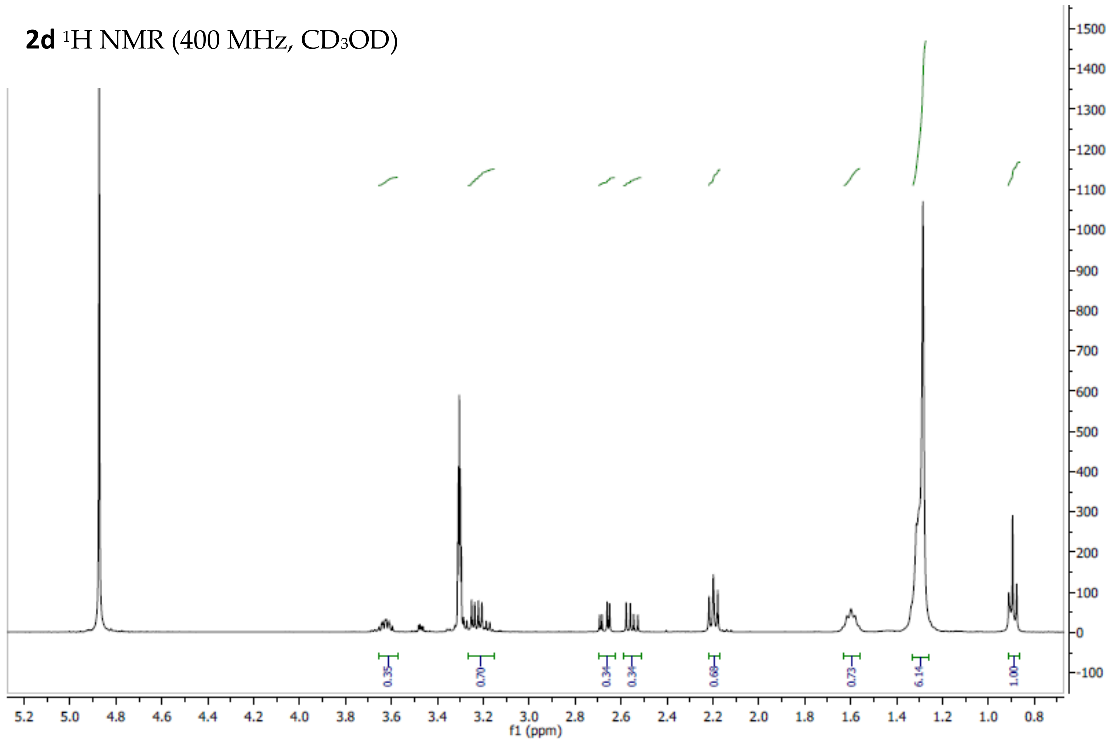

2d ^{13}C NMR (101 MHz, CD_3OD)

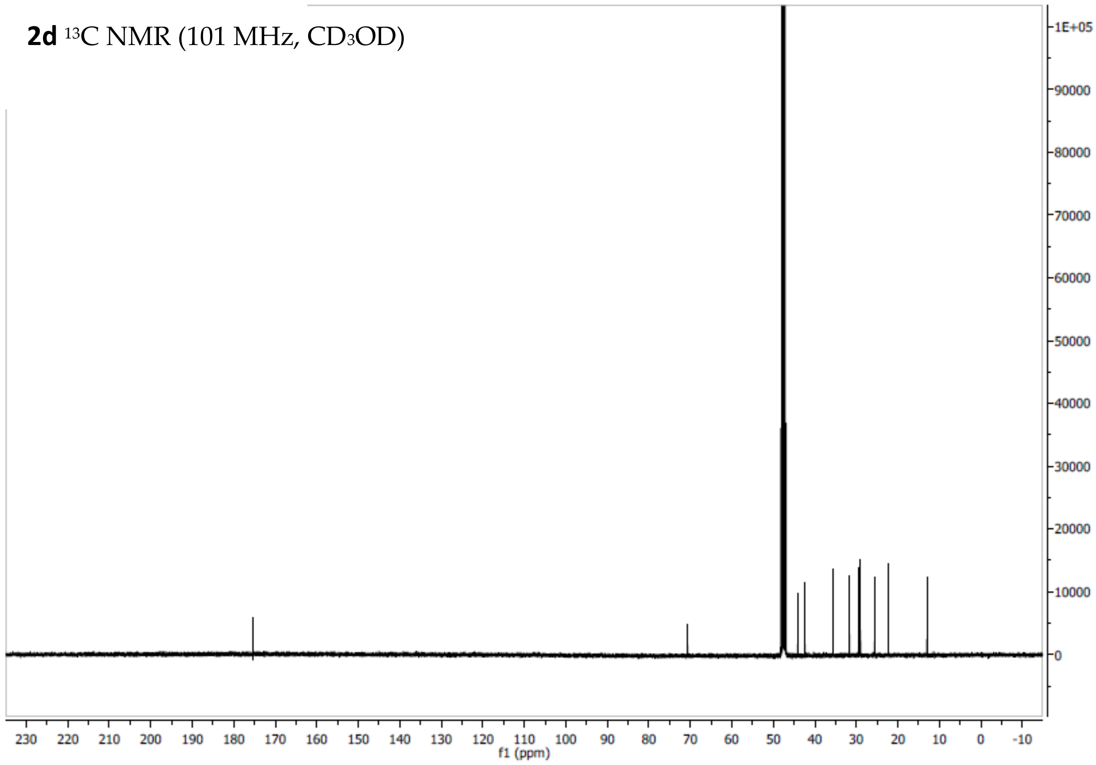

2e ^{1}H NMR (400 MHz, $CDCl_3$)

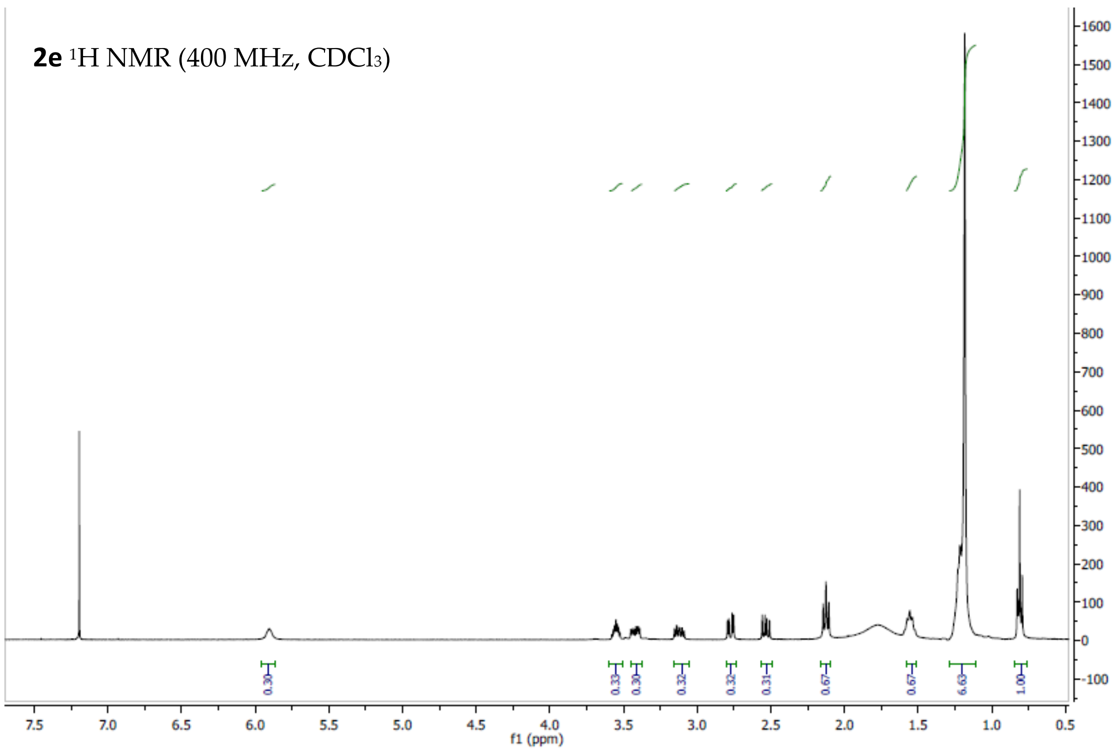

2e ^{13}C NMR (101 MHz, $CDCl_3$)

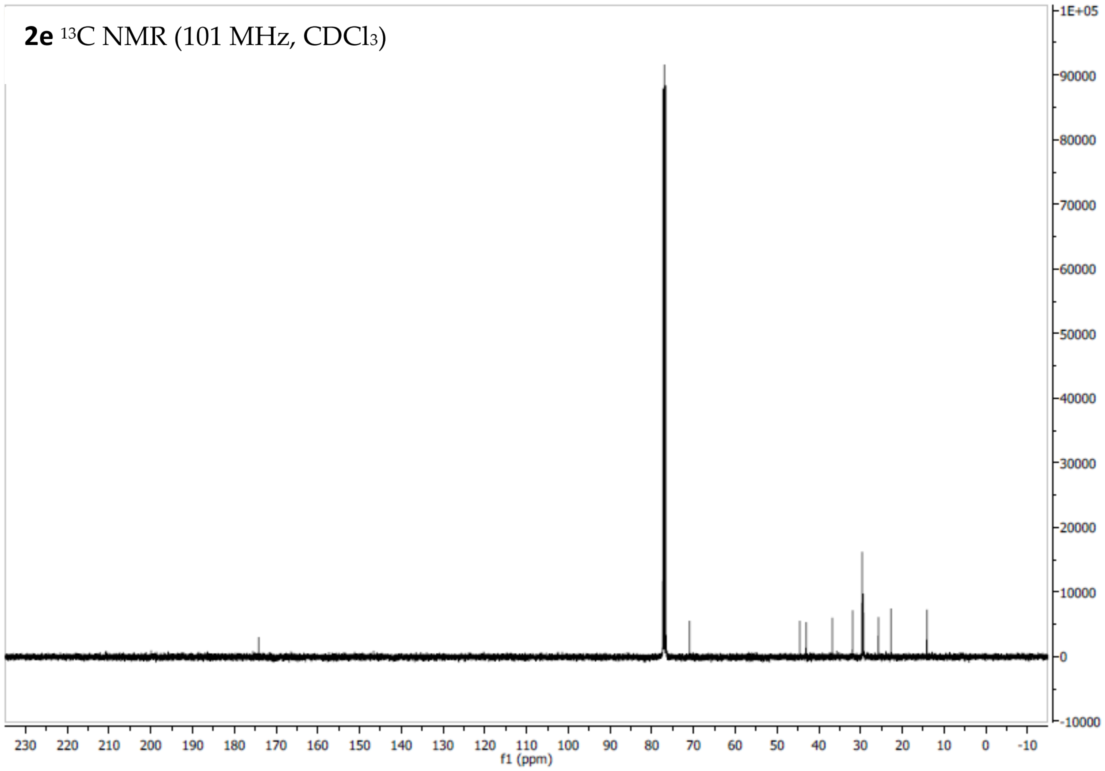

2g ^{1}H NMR (400 MHz, $CDCl_3$)

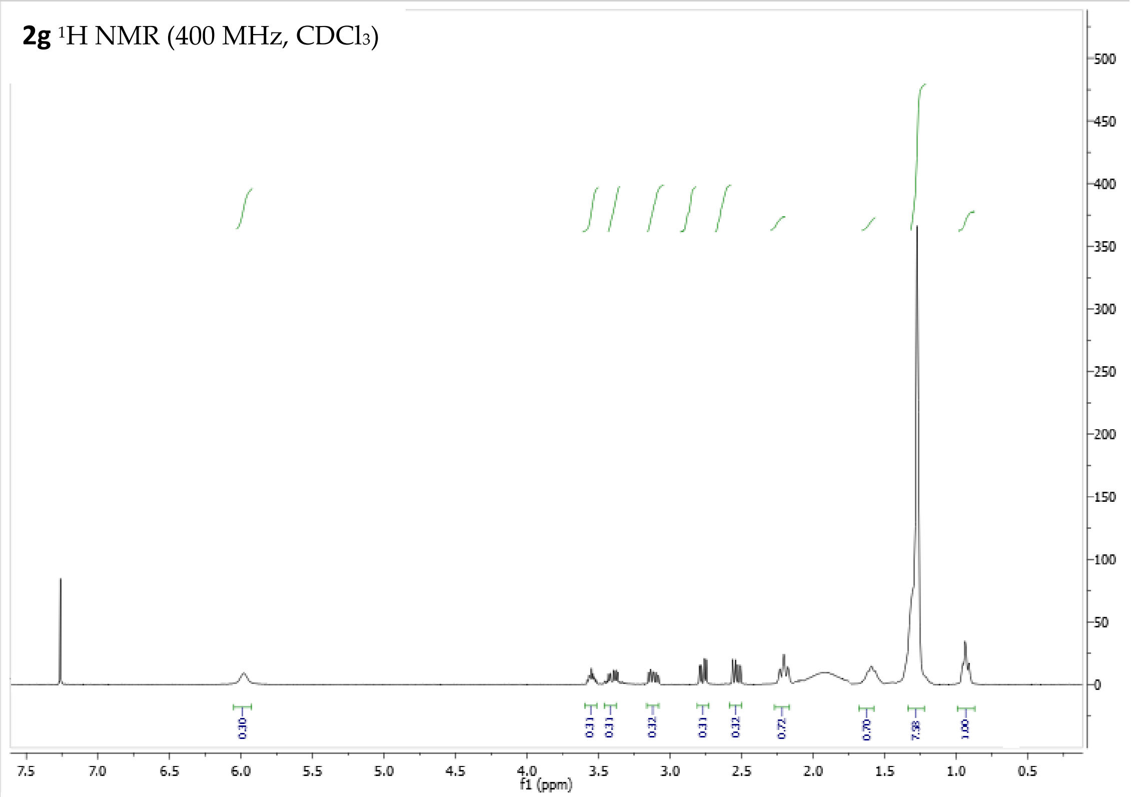

2g ^{13}C NMR (101 MHz, $CDCl_3$)

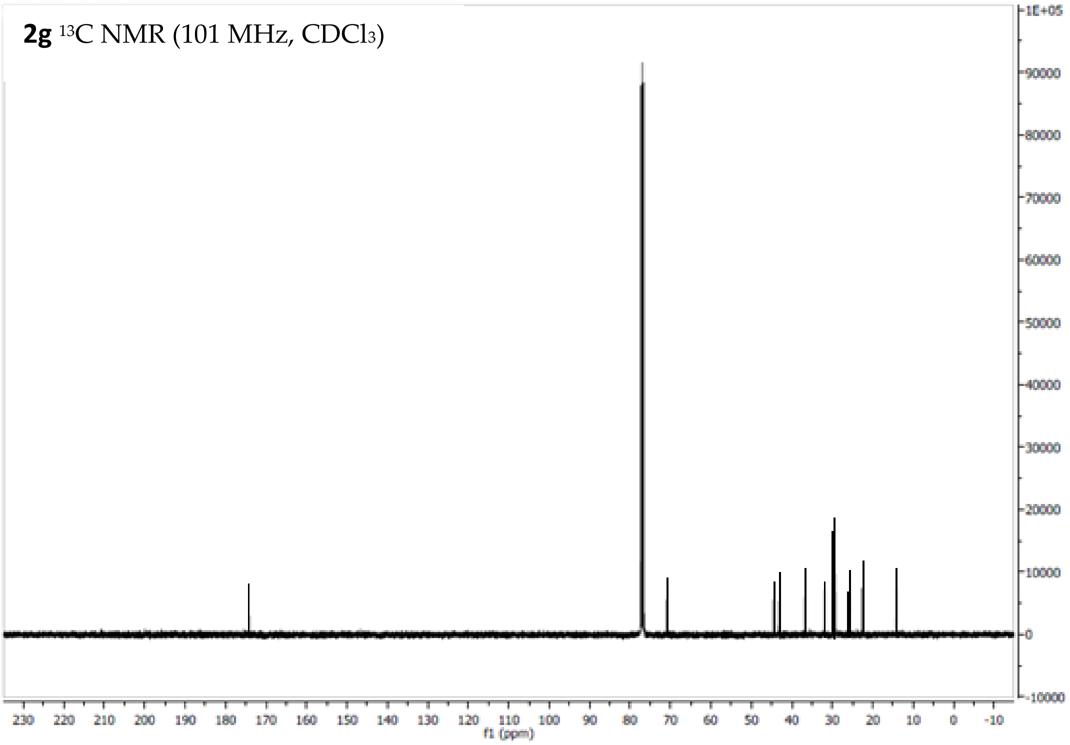

2h (^{1}H NMR (400 MHz, CD_3OD)

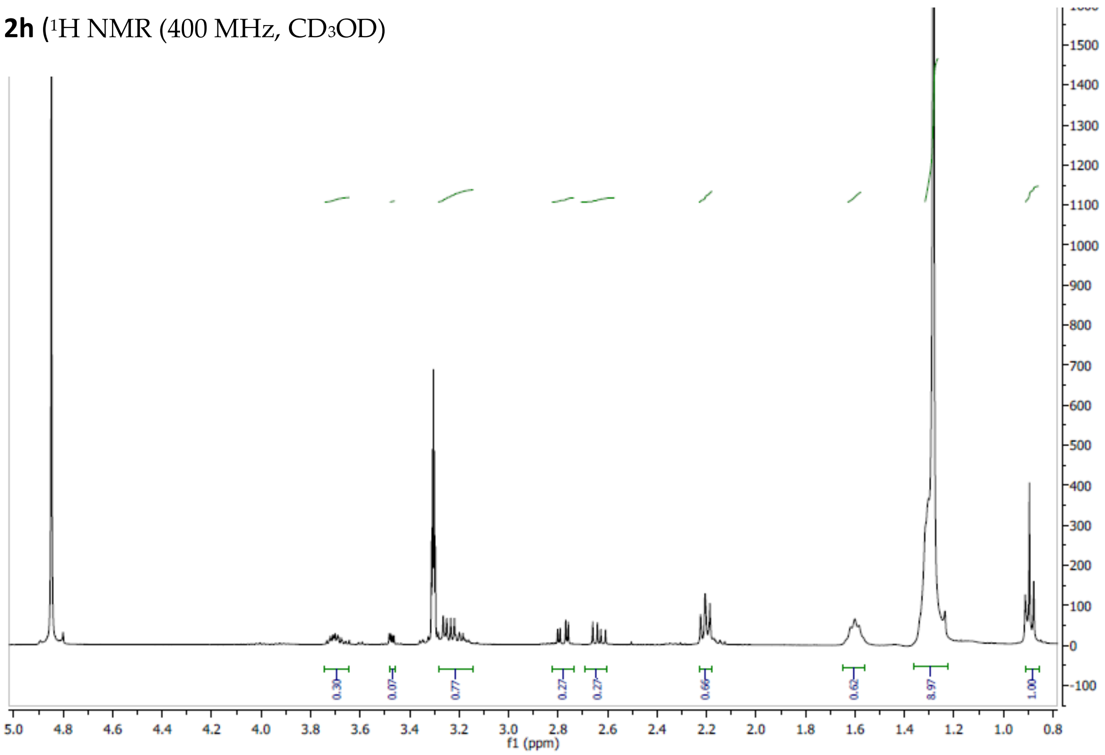

2h ^{13}C NMR (101 MHz, CD_3OD

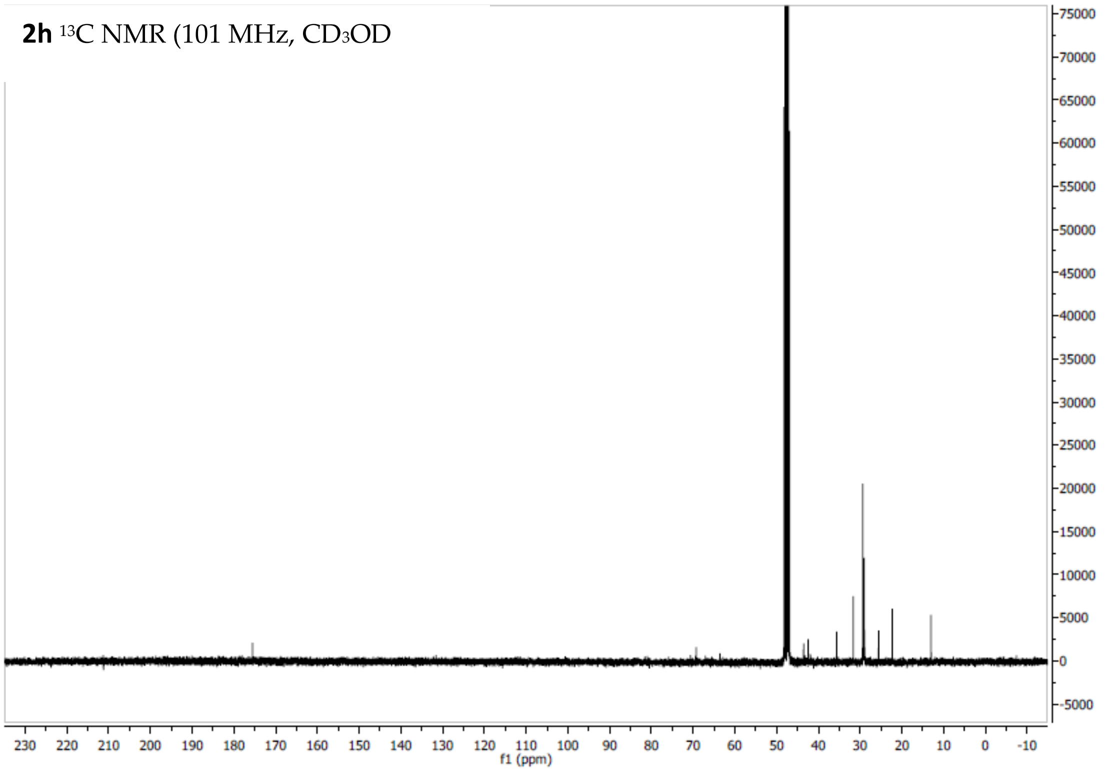

2i ^{1}H NMR (400 MHz, CD_3OD)

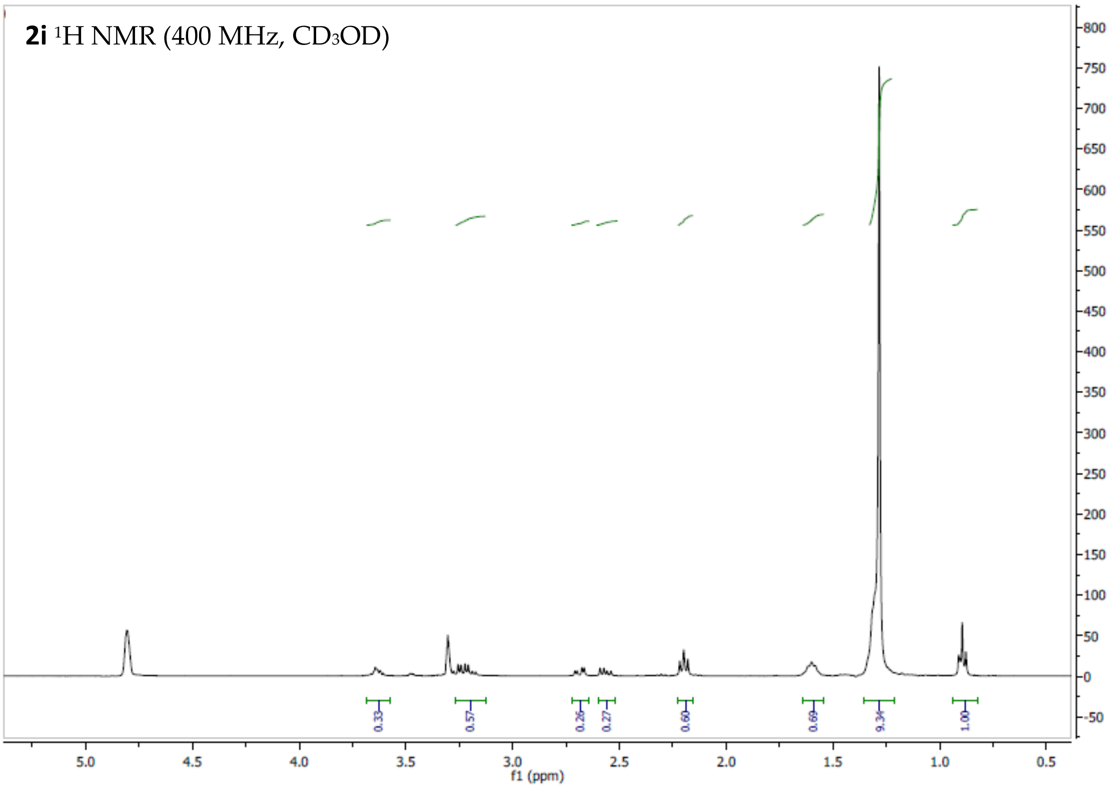

2i ^{13}C NMR (101 MHz, CD_3OD)

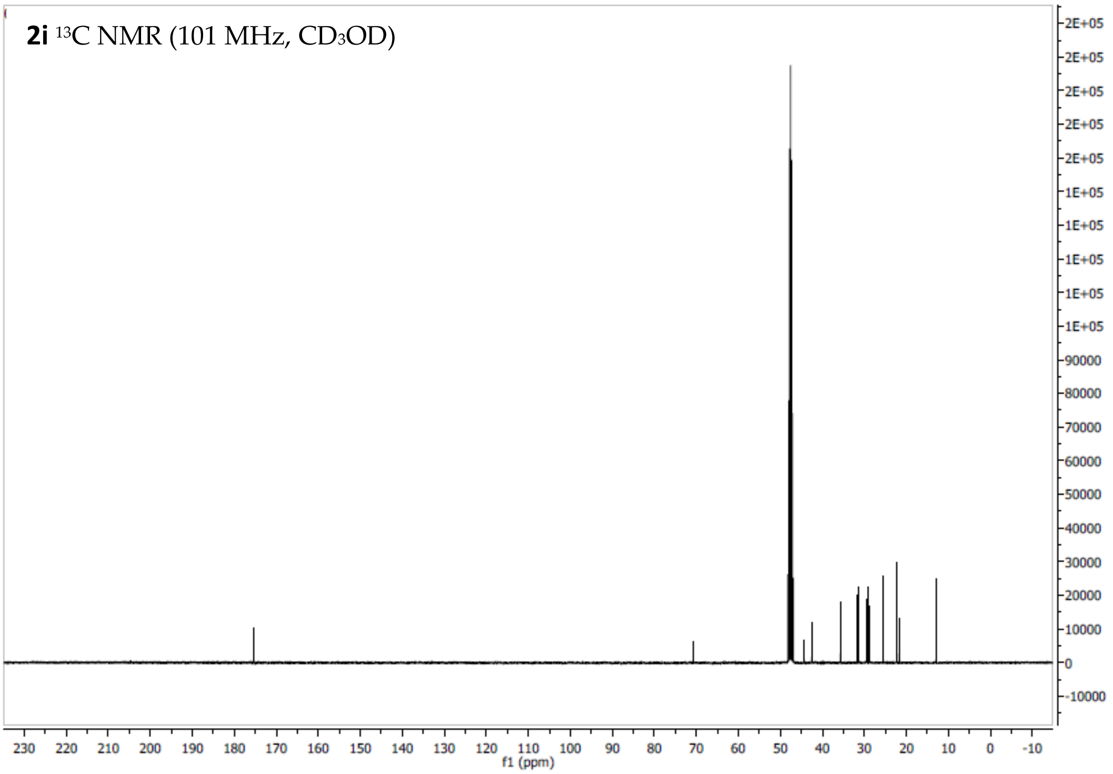

Supplement: Supplementary file 1 [file molecules-24-03777-s001.pdf]
